# Ulcerative Colitis Remission Status After Induction With Mesalazine Predicts Maintenance Outcomes: the MOMENTUM Trial

**DOI:** 10.1093/ecco-jcc/jjw049

**Published:** 2016-02-23

**Authors:** David T. Rubin, Marc Bradette, Libor Gabalec, Daniela Dobru, Juan Márquez, Susi Inglis, Elizabeth Magee, Dory Solomon, Geert D’Haens, on behalf of the Ulcerative Colitis Remission Study Group

**Affiliations:** ^a^University of Chicago Medicine, Inflammatory Bowel Disease Center, Chicago, IL, USA; ^b^CHUQ Research Center, L’Hôtel-Dieu de Québec, Québec, Canada; ^c^Orlickoustecka Nemocnice a.s., Department of Internal Medicine, Ústí nad Orlicí, Czech Republic; ^d^University of Medicine and Pharmacy Târgu Mures, Gastroenterology Department, Târgu Mures, Romania; ^e^Department of Colorectal Surgery, Clínica Las Américas, Medellin, Columbia; ^f^Shire, Basingstoke, UK; ^g^Shire, Wayne, PA, USA; ^h^Academic Medical Centre, Department of Gastroenterology and Hepatology, Amsterdam, The Netherlands

**Keywords:** 5-aminosalicylic acid [5-ASA], Inflammatory bowel disease, MOMENTUM

## Abstract

**Background and Aims:**

This study assessed the efficacy of maintenance treatment with multimatrix mesalazine following achievement of complete or partial remission after induction treatment with high-dose multimatrix mesalazine.

**Methods:**

In this phase 3b/4, open-label, multicentre, prospective, single-arm study, patients with mild-to-moderate ulcerative colitis were treated with multimatrix mesalazine 4.8g/day once daily for 8 weeks [induction phase]. At Week 8, those who achieved complete or partial remission, based on predefined clinical and endoscopic criteria, were eligible to receive 12 months of multimatrix mesalazine 2.4g/day once daily maintenance therapy. The primary endpoint was the proportion of patients in complete remission at Month 12.

**Results:**

A total of 717 patients received induction treatment; 25.9% and 39.3% of patients achieved complete and partial remission, respectively, at Week 8. A total of 461 patients entered the maintenance phase. The likelihood of remaining in/achieving complete remission at Month 12 was higher for patients who entered the maintenance phase in complete remission compared with those who began maintenance in partial remission [47.8% vs 26.0%; *p* < 0.001]. At Month 12, mucosal healing [endoscopy score ≤ 1] was demonstrated in 76.4% [139/182] and 63.5% [176/277] of those who were in complete and partial remission, respectively, at the end of induction.

**Conclusion:**

Patients achieving complete remission before dose reduction were more likely to remain in remission at Month 12.

## 1. Introduction

Ulcerative colitis [UC] is an inflammatory disease of the large bowel characterised by relapsing and/or remitting gastrointestinal and systemic symptoms, which include bloody diarrhoea and rectal urgency.^1,2^ Primary goals of UC management are induction and maintenance of disease remission, including healing of the mucosa, to improve patients’ health and quality of life.^3,4^ With no known cure for UC, most patients will need lifelong maintenance medical therapy to help prevent disease relapse.^4,5^ In a prospective trial by Meucci and colleagues,^6^ patients with mild-to-moderate UC relapse received induction treatment with a combination of oral and topical mesalazine. If patients were in clinical *and* endoscopic remission [Mayo score ≤ 1], the observed 1-year incidence of relapse was 23% compared with a relapse rate of 80% in patients who attained only clinical, but not endoscopic, remission. Similarly, in patients with moderate-to-severe UC relapse who were treated with infliximab in the Active Ulcerative Colitis Trial [ACT]-1 and ACT-2,^7^ colectomy risk was significantly lower if patients had a Mayo endoscopy subscore ≤ 1 than if the mucosa was more severely affected [Mayo score ≥ 2].

For patients with active mild-to-moderate UC, mesalazine [5-aminosalicylic acid; 5-ASA] has an established efficacy and a favourable safety profile; therefore, it has been recommended as first-line therapy.^3,8^ Multimatrix mesalazine is a once-daily [QD], oral formulation of 5-ASA for induction and maintenance of remission in mild-to-moderate UC.^9^ In two phase 3, placebo-controlled, double-blind, randomised studies, multimatrix mesalazine was shown to be safe and effective in the induction of complete [clinical and endoscopic] remission.^10,11^ A subsequent long-term maintenance study of multimatrix mesalazine treatment demonstrated high clinical and endoscopic remission rates in a subset of patients from the combined phase 3 induction studies who had achieved complete [clinical and endoscopic] remission.^12^ A phase 4, open-label study also demonstrated efficacy of multimatrix mesalazine in maintaining quiescence in patients with UC,^13^ and another phase 3 study showed that multimatrix mesalazine was not inferior to a twice-daily [BID], delayed-release formulation of mesalazine in endoscopic maintenance of remission.^14^

Although multimatrix mesalazine has demonstrated efficacy in induction and maintenance of remission in UC, little is currently known about the effect of the success of induction treatment on long-term outcomes. In addition, dose reduction during UC maintenance is frequently employed as a therapeutic approach to reduce the number of pills patients have to take, or it can merely be a consequence of poor adherence. This strategy has not been formally studied in a prospective fashion. This study [MOMENTUM trial; ClinicalTrials.gov Identifier: NCT01124149] was designed to determine whether patients who achieved complete remission with mucosal healing after induction therapy with multimatrix mesalamize had better long-term outcomes compared with those who attained only partial remission.

## 2. Methods

### 2.1. Patients

Participants were aged ≥ 18 years and had an established diagnosis of UC by earlier sigmoidoscopy or colonoscopy with compatible histology, or were newly diagnosed with UC. Disease activity was assessed using a modified UC–Disease Activity Index [UC-DAI]. The UC-DAI consists of 4 parameters [each scored from 0–3; maximum score = 12]: rectal bleeding, stool frequency, rectosigmoidoscopy, and Physician’s Global Assessment [PGA]. The standard UC-DAI was modified so that an endoscopy score of 1 [mild disease] did not include friability, which was scored as 2 [moderate disease], as previously described.^10,11^ Eligible patients had active disease [total modified UC-DAI of 4 to 10], with an endoscopy score ≥ 1 and PGA ≤ 2. Patients could receive stable maintenance therapy of 5-ASA ≤ 3.2g/day [excluding multimatrix mesalazine] or the equivalent dose of sulphasalazine. This treatment was discontinued at study start. Pregnant women were excluded. Additional exclusion criteria included: onset of flare on mesalazine > 6 weeks before the study [no time limit for flare onset if untreated], Crohn’s disease, proctitis [inflammation extent ≤ 15cm from anus], bleeding disorders, active peptic ulcer disease, asthma and known hypersensitivity to mesalazine, previous colonic surgery, moderate/severe renal and/or hepatic impairment, and previous biologic [eg anti–tumour necrosis factor] use. Stool cultures were performed during screening and, if positive for enteric pathogens, the patient was ineligible.

The study protocol, informed consent document, and all patient recruitment information were approved by the institutional review board or independent ethics committee at each site. The study was conducted in accordance with International Conference on Harmonisation Good Clinical Practice guidelines and local ethical and legal regulations, in line with the principles of the Declaration of Helsinki. All authors had access to study data, and reviewed and approved the final manuscript.

### 2.2. Study design

**Figure 1.**
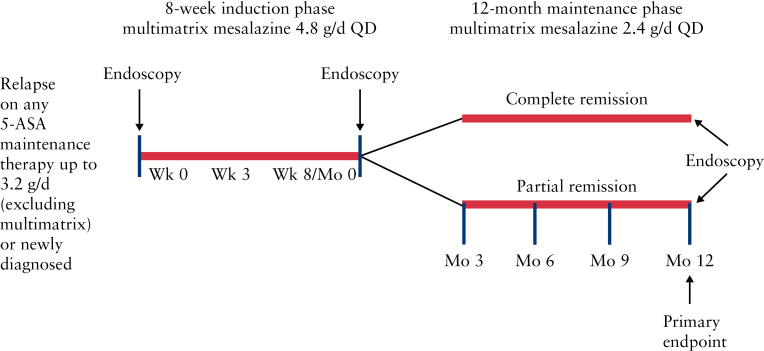
Study design. QD, once daily; 5-ASA, 5-aminosalicylic acid.

In this phase 3b/4, open-label, multicentre, single-arm, prospective study, all enrolled patients were treated with multimatrix mesalazine 4.8g/day QD for 8 weeks in the induction phase [Figure 1]. Patients achieving complete remission or partial remission [defined in the Study objectives section] by Week 8 were eligible to continue with 12 months of multimatrix mesalazine 2.4g/day QD maintenance treatment. The study was conducted at 83 sites across 14 countries, between June 2010 and December 2012.

Study visits occurred at screening; baseline [Week 0 of induction]; Week 3 of induction; Week 8 of induction [Month 0 of maintenance]; and at Months 3, 6, 9, and 12 of maintenance. Endoscopies with biopsies were performed at screening or baseline, Week 8/Month 0, and Month 12. Endoscopies were performed by the same investigator/endoscopist for scoring consistency, but central independent reading was not used. Treatment compliance was measured by tablet count and was calculated for all study visits after baseline visit; patients were considered compliant if they had taken 80–120% of the prescribed/dispensed medication at each visit.

### 2.3. Study objectives

The primary objective of this study was to compare proportions of patients in complete remission [defined as in previous studies of multimatrix mesalazine as a modified UC-DAI score ≤ 1, with a score of 0 for both rectal bleeding and stool frequency, and a ≥ 1-point reduction in endoscopy score from baseline]^10,11,15^ following 12 months of maintenance therapy, between those who achieved complete remission by Week 8 of induction treatment vs those who achieved partial remission [modified UC-DAI ≤ 3, with a combined stool frequency and rectal bleeding score ≤ 1, and not in complete remission] after induction.

Secondary objectives from the induction phase included assessment of the proportion of patients in complete remission at Week 8 and assessment of symptom improvement [≥ 1-point reduction in rectal bleeding and stool frequency scores] at Weeks 3 and 8. Safety and tolerability during both induction and maintenance phases were also examined.

Secondary endpoints that were studied in the maintenance phase, again comparing patients with complete and partial remission by Week 8, included: 1] the proportion of patients in each group who achieved clinical remission [stool frequency and rectal bleeding scores of 0] at Month 12 regardless of endoscopy, 2] the proportion of patients in each group who achieved or maintained mucosal healing [endoscopy score ≤ 1 on the UC-DAI] at Month 12, and 3] the time to relapse in each group [defined as the need for treatment escalation for UC, including colectomy, or study withdrawal due to lack of efficacy].

### 2.4. Statistical analyses

Based on remission data from previous studies,^10,11,12,15^ it was assumed that the proportion who would be in complete remission at Month 12 would be 65% for patients in complete remission at Month 0, and 50% for patients in partial remission at Month 0. With 80% power and a ratio of patients in complete remission to partial remission of 1.5:1, 382 patients were estimated to be necessary for inclusion in the maintenance phase. With the assumption that 55% of enrolled patients would continue into maintenance, 695 patients needed to be enrolled; it was anticipated that ~ 875 patients would need to be screened to enroll the required 695 patients into the study, and that 306 patients would complete through Month 12 of maintenance.

Analyses of efficacy endpoints were performed on the respective efficacy populations for the induction and maintenance phases, defined as patients who received ≥ 1 dose of investigational drug and had ≥ 1 post-dose efficacy assessment in either treatment phase. The primary endpoint analysis was performed on the maintenance phase efficacy population; all patients who did not complete the study [missing data at Month 12] were assumed not to be in remission. A sensitivity analysis of the primary endpoint was also conducted at final on-treatment assessment [FoTA], defined as data from Month 12 or an early withdrawal visit. A second sensitivity analysis of the maintenance data was conducted on the patient population, with biopsies that confirmed histologically active UC at baseline [maintenance phase positive baseline biopsy population]. For these analyses, the proportion of patients in complete remission at Month 12 were compared between those who had achieved complete vs partial remission during induction, using a logistic regression model with a term for remission group only.

The proportion of patients in clinical remission at Month 12 and FoTA was compared between the complete and partial remission cohorts at Month 0 for the maintenance phase efficacy population and the maintenance phase positive baseline biopsy population; these comparisons were performed using the same logistic regression model as the primary analysis. The proportions of patients who achieved or maintained mucosal healing at Month 12 and at FoTA were summarised by remission group.

Adverse event [AE] summaries for the induction and maintenance phases were conducted on all patients who had ≥ 1 dose of study drug [safety population] in that phase. Statistical programming and analyses of the efficacy and safety populations were performed using SAS^®^ Version 9.1 [SAS Institute, Cary, NC, USA] or higher.

## 3. Results

### 3.1. Patients

**Figure 2.**
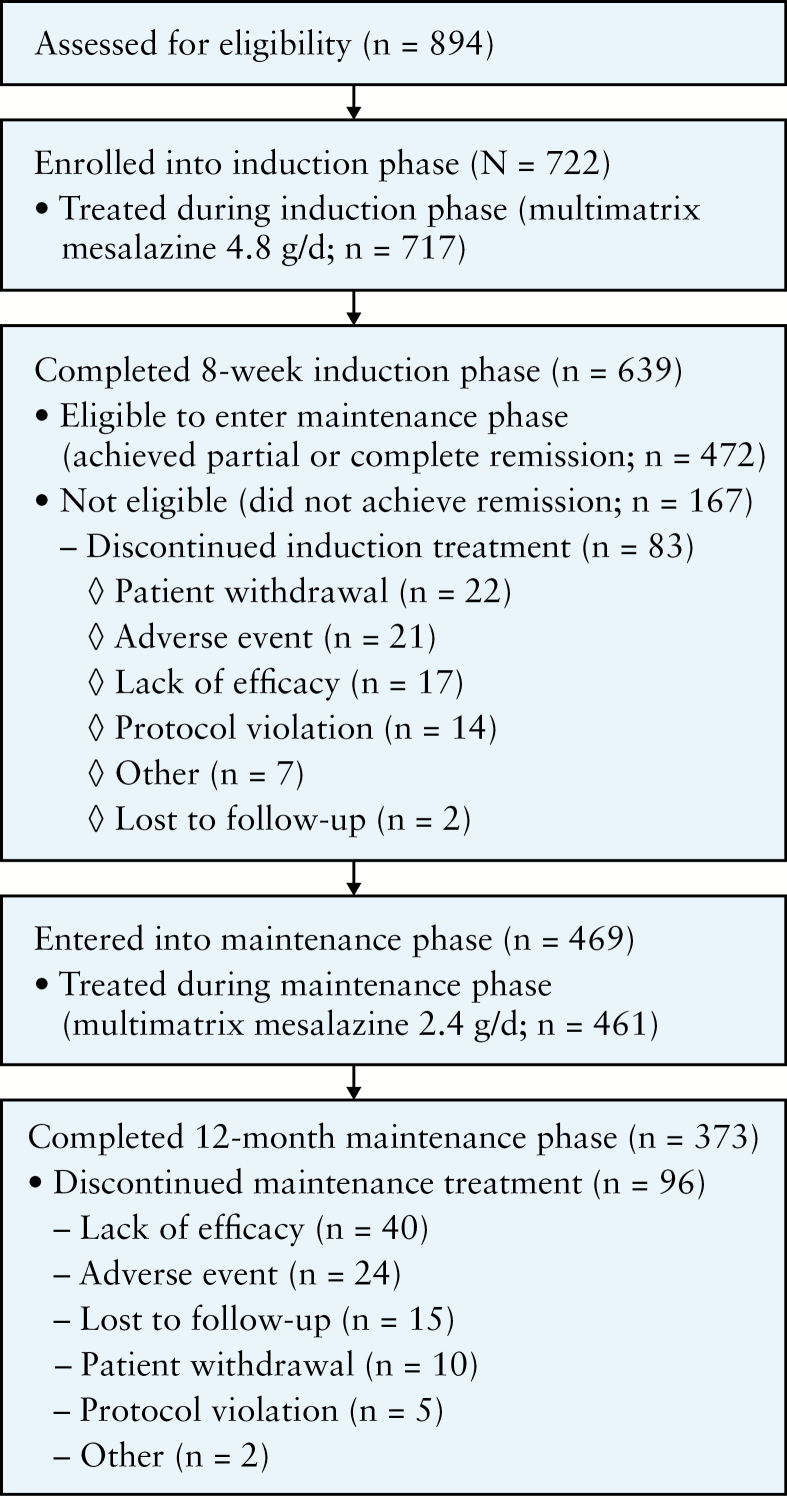
Patient flow diagram.

A total of 722 patients were enrolled from June 2010 to December 2012; 717 [99.3%] patients were treated and 639 [88.5%] patients completed the 8-week induction phase [Figure 2]. The most common reasons for early study discontinuation in the induction phase were patient withdrawal [3.0%], AEs [2.9%], and lack of efficacy [2.4%]. A total of 472 [65.4%] patients were eligible to enter the maintenance phase, of whom 469 agreed to participate; patients who did not achieve either partial or complete remission were excluded. Maintenance treatment was initiated in 461 [98.3%] patients, of whom 373 [79.5%] completed the 12-month maintenance phase. The most common reasons for early withdrawal in the maintenance phase included lack of efficacy [8.5%] and AEs [5.1%]. Treatment non-compliance was very low in both phases: 2.0% and 0.9% of patients in the induction and maintenance phases, respectively, consumed < 80% of their prescribed medication. Baseline patient demographic and clinical characteristics are presented in Table 1; patients in the two maintenance groups [those with complete remission and those with partial remission following induction] were comparable with regard to baseline characteristics. A total of 49.7% of enrolled patients had previous treatment with mesalazine, and < 1 % had previous treatment with corticosteroids.

**Table 1.** Baseline patient demographic and clinical characteristics^a^

Characteristic, *n* [%]	Induction phaseOverall [*N* = 717]	Maintenance phase^b^Overall [*N* = 461]
Age, mean [SD], years	42.9 [13.97]	42.7 [14.26]
Sex		
Male	409 [57.0]	260 [56.4]
Female	308 [43.0]	201 [43.6]
Race		
White	428 [59.7]	256 [55.5]
Non-white	289 [40.3]	205 [44.5]
Black/African American	10 [1.4]	7 [1.5]
Asian	206 [28.7]	143 [31.0]
American Indian/Alaska Native	13 [1.8]	12 [2.6]
Other	60 [8.4]	43 [9.3]
UC history		
How was UC first established?^c^		
Sigmoidoscopy	260 [36.3]	179 [38.8]
Colonoscopy	566 [78.9]	357 [77.4]
Barium enema	18 [2.5]	11 [2.4]
Compatible histology	713 [99.4]	460 [99.8]
Full extent of disease > 15 cm	714 [99.6]	461 [100]
Classification of extent of disease		
Left-sided	557 [77.7]	359 [77.9]
Involvement of transverse colon	50 [7.0]	33 [7.2]
Pancolitis	110 [15.3]	69 [15.0]
Rectal involvement	653 [91.1]	420 [91.1]
Mean [SD] time since current acute episode, days^c^	38.6 [179.22]	42.4 [218.61]
Number of newly diagnosed patients	70 [9.8]	38 [8.2]
Mean [SD] duration since diagnosis, months^d,e^	71.2 [86.56]	69.8 [80.49]
Number of acute episodes in the past year^e^		
0	72 [10.0]	50 [10.8]
1–2	479 [66.8]	320 [69.4]
3–4	86 [12.0]	48 [10.4]
5–6	4 [0.6]	2 [0.4]
≥ 7	4 [0.6]	1 [0.2]
Missing	2 [0.3]	2 [0.4]
Number of acute episodes since diagnosis^e^		
0	16 [2.2]	10 [2.2]
1–2	247 [34.4]	164 [35.6]
3–4	177 [24.7]	115 [24.9]
5–6	74 [10.3]	51 [11.1]
≥ 7	125 [17.4]	79 [17.1]
Missing	8 [1.1]	4 [0.9]
Mean [SD] duration of past acute episode, days^e^	32.7 [37.47]	33.5 [41.71]

SD, standard deviation; UC, ulcerative colitis.

^a^Safety populations for induction and maintenance phases, respectively.

^b^Demographic and baseline characteristics were similar in the maintenance phase between patients in complete remission at Month 0 and patients in partial remission at Month 0.

^c^Multiple procedures may have been used to establish first diagnosis of UC.

^d^Relative to screening.

^e^Only for patients who were not newly diagnosed.

### 3.2 Efficacy

#### 3.2.1. Induction phase with multimatrix mesalazine 4.8g/day

At Week 8, 25.9% [186/717] of patients achieved complete remission and 39.3% [282/717] achieved partial remission. Rectal bleeding scores improved by ≥ 1 point in 42.4% of patients by Week 3 and 59.8% by Week 8. Stool frequency scores improved by ≥ 1 point in 38.5% and 58.9% of patients by Weeks 3 and 8, respectively. By Weeks 3 and 8, the combination of both rectal bleeding and stool frequency scores showed improvement from baseline in 25.2% and 45.3% of patients, respectively.

#### 3.2.2 Maintenance phase primary endpoint: complete remission at Month 12 with multimatrix mesalazine 2.4g/day

Of 182 patients in complete remission at Month 0 of the maintenance phase, 87 [47.8%] remained in complete remission at Month 12; of 277 patients in partial remission at Month 0, 72 [26.0%] achieved complete remission at Month 12. The odds ratio [OR] of complete remission to partial remission was 2.61 (95% confidence interval [CI]: 1.76, 3.87), and the difference between the two groups was statistically significant [*p* < 0.001].

Sensitivity analyses were consistent with the primary endpoint analysis. At FoTA, 48.9% of patients in complete remission at Month 0 were in complete remission at Month 12, compared with 26.4% in partial remission at Month 0 [OR, 2.67; 95% CI: 1.80, 3.97; *p* < 0.001]. For the maintenance phase positive baseline biopsy population, 45.3% of patients in complete remission at Month 0 were in complete remission at Month 12, compared with 25.0% in partial remission at Month 0 [*p* < 0.001].

#### 3.2.3. Secondary endpoints

Looking at clinical remission only, 58.8% [107/182] of patients who had been in complete remission at Month 0 were in clinical remission at Month 12, compared with 40.4% [112/277] of patients in partial remission at Month 0 [*p* < 0.001]. The difference between groups for clinical remission was smaller than that for complete remission, where endoscopy is included in the remission definition. Sensitivity analyses at FoTA and for the maintenance phase positive baseline biopsy population were consistent with this result.

**Figure 3.**
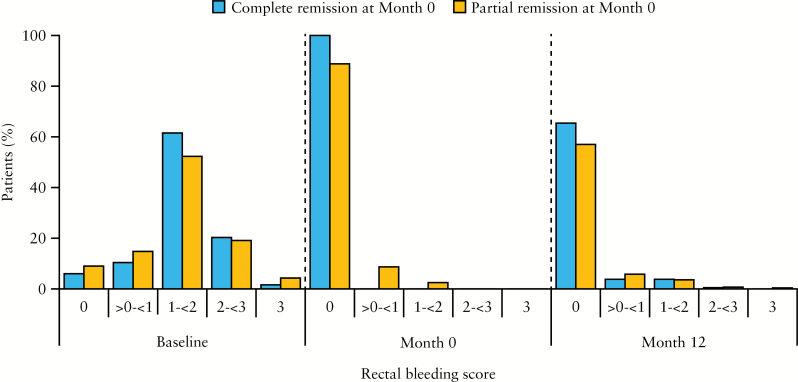

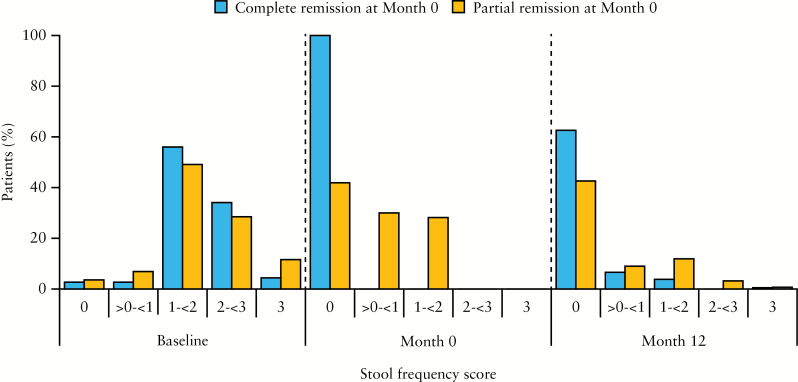
[A] Rectal bleeding scores and [B] stool frequency scores at baseline, Month 0, and Month 12 among patients who achieved complete or partial response at Month 0; maintenance phase efficacy population [*n* = 459]. Percentages at Month 12 do not add up to 100% due to the absence of scores from patients who discontinued the study before Month 12.

Symptom scores at baseline, Month 0, and Month 12 are shown in Figure 3. For those in complete remission at Month 0, by definition, 100% had rectal bleeding and stool frequency scores of 0 at Month 0; at Month 12, 65.4% and 62.6% of these patients, respectively, had maintained rectal bleeding and stool frequency scores of 0. For those in partial remission at Month 0, 88.8% and 41.9% of patients, respectively, had rectal bleeding and stool frequency scores of 0 at Month 0; and at Month 12, 57.0% and 42.6% of this group had rectal bleeding and stool frequency scores of 0.

**Figure 4.**
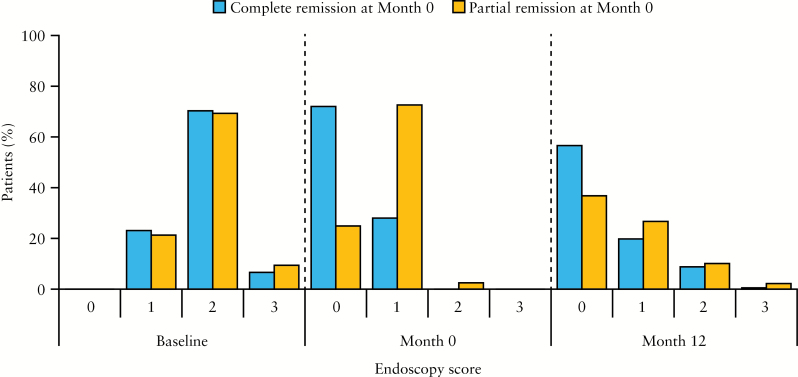
Endoscopy scores at baseline, Month 0, and Month 12 among patients who achieved complete or partial response at Month 0; maintenance phase efficacy population [*n* = 459]. Percentages at Month 12 do not add up to 100% due to the absence of scores from patients who discontinued the study before Month 12.

The proportions of patients with mucosal healing [endoscopy score ≤1] at Month 12 were 76.4% and 63.5%, respectively, for those in complete vs partial remission at Month 0 [nominal *p* = 0.0037]. Endoscopy scores after 12 months of maintenance therapy with multimatrix mesalazine are shown in Figure 4. Relapse rates at Month 12 were 6.0% and 10.5%, respectively, for those in complete and partial remission at Month 0. Among the patients who had relapsed, the median time to relapse was 176 days for those who achieved complete remission at Month 0 [*n* = 11], and 148 days for patients in partial remission at Month 0 [*n* = 29].

### 3.3 Safety

During induction, 202 [28.2%] patients experienced ≥ 1 treatment-emergent AE [TEAE]. The most commonly reported TEAE was headache [2.1%]. In the maintenance phase, TEAEs were less frequent in the complete vs partial remission group. Overall, 68/183 [37.2%] patients in complete remission at Month 0 and 139/278 [50.0%] patients in partial remission at Month 0 reported ≥ 1 TEAE [Table 2]. The most common TEAEs during maintenance were UC, headache, bronchitis, influenza, nasopharyngitis, and back pain. The frequency of TEAEs was generally low, and most TEAEs were mild or moderate in severity.

**Table 2.** TEAEs reported in the maintenance phase^a^

Event, *n* [%]	Complete remission at Month 0 [*n* = 183]	Partial remission at Month 0 [*n* = 278]
Any TEAE	68 [37.2]	139 [50.0]
Any severe TEAE	3 [1.6]	12 [4.3]
Any serious TEAE	4 [2.2]	10 [3.6]
Any drug-related TEAE	7 [3.8]	24 [8.6]
Any serious drug-related TEAE	0	1 [0.4]
Any TEAE leading to drug discontinuation	15 [8.2]	42 [15.1]
Any TEAE leading to death	0	1 [0.4]
TEAEs reported in > 1% of patients		
Ulcerative colitis	14 [7.7]	29 [10.4]
Headache	6 [3.3]	10 [3.6]
Bronchitis	6 [3.3]	1 [0.4]
Nasopharyngitis	5 [2.7]	6 [2.2]
Influenza	3 [1.6]	8 [2.9]
Diarrhoea	3 [1.6]	5 [1.8]
Pyrexia	3 [1.6]	5 [1.8]
Pharyngitis	3 [1.6]	4 [1.4]
Rectal bleeding	3 [1.6]	3 [1.1]
Abdominal pain	3 [1.6]	2 [0.7]
Hypertension	3 [1.6]	1 [0.4]
Back pain	2 [1.1]	6 [2.2]
Frequent bowel movements	2 [1.1]	4 [1.4]
Herpes zoster	2 [1.1]	0
Osteoarthritis	2 [1.1]	0
Cough	2 [1.1]	0
Anaemia	1 [0.5]	5 [1.8]
Arthralgia	1 [0.5]	4 [1.4]
Urinary tract infection	1 [0.5]	3 [1.1]
Sinusitis	0	4 [1.4]
Upper respiratory tract infection	0	3 [1.1]
Uveitis	0	3 [1.1]
Aphthous stomatitis	0	3 [1.1]
Toothache	0	3 [1.1]
Rash	0	3 [1.1]

TEAE, treatment-emergent adverse event.

^a^Safety population.

During induction, 47 patients experienced 61 TEAEs that led to study withdrawal; the events most frequently leading to withdrawal were lack of efficacy [*n* = 24; 3.3%], UC [*n* = 9; 1.3%], and diarrhoea [*n* = 3; 0.4%]. In the maintenance phase, the proportion of patients experiencing TEAEs leading to withdrawal was almost twice as high in the partial remission group [15.1%] than in the complete remission group [8.2%]. For those in partial remission at Month 0, the events most frequently leading to withdrawal were lack of efficacy [*n* = 18; 6.5%] and UC [*n* = 17; 6.1%]; for those in complete remission at Month 0, the most frequently reported TEAE leading to withdrawal was UC [*n* = 10; 5.5%].

During induction, 13 [1.8%] patients reported 14 serious TEAEs. Three were considered by the investigator to be related to the study drug: UC exacerbation [*n* = 1], acute pancreatitis [*n* = 1], and lung infection [*n* = 1]. These patients were discontinued from the study and the events subsequently resolved. During maintenance, 14 patients experienced 17 serious TEAEs, of which 1 [worsening diarrhoea] was considered related to the study drug. The event resolved and the patient completed the study. One patient died following a cerebrovascular incident during the maintenance phase, which was considered unrelated to the study drug.

## 4. Discussion

This prospective study is the first to examine the scheduled dose reduction of 5-ASA in UC patients. We explored maintenance outcomes in patients with UC treated with an induction regimen of multimatrix mesalazine 4.8g/day, then switched to maintenance phase at 2.4g/day. The primary endpoint was met, as a significantly higher proportion of patients in complete vs partial remission at the start of the maintenance phase were in complete remission 12 months later. These results were supported by sensitivity analyses on the primary endpoint, as well as results from the maintenance phase secondary endpoint analyses.

Our study indicates that the outcomes following mesalazine induction are predictive of long-term outcome. Half of the patients who achieved complete remission during induction with high-dose multimatrix mesalazine were able to maintain complete remission after 12 months of treatment with a reduced dosage, a success rate almost twice as high as in the patients entering maintenance in partial remission. Nonetheless, one in four patients attaining partial remission after induction reached complete remission 12 months later. In addition, > 40% of patients in the partial remission group had symptom scores of 0 at Month 12, and > 60% of patients in the partial remission group demonstrated mucosal healing at Month 12. It is possible that some of the observed difference between the groups is due to overlap of bowel irritability; this is a common challenge in symptom-based assessments of disease activity in UC. However, non–symptom-based measures [eg, endoscopy] also resulted in significant group differences, suggesting that underlying inflammation was a primary factor in differentiation. These findings highlight the importance of long-term mesalazine treatment for patients with UC and suggest that, contrary to previous and non–evidence-based assertions, 2.4g/day of multimatrix mesalazine during maintenance may be beneficial for both those in complete and those in partial remission, following induction at higher doses.

It is well appreciated that both patients and physicians dose reduce mesalazine while in maintenance phase. This study showed that complete remission was a significant predictor of successful dose reduction in the maintenance phase; patients attaining that endpoint may be most suitable for this strategy. Conversely, patients without complete remission, including mucosal healing, would probably benefit from an additional period of high-dose mesalazine treatment, additional topical treatment, or even escalation to other classes of therapies. These patients may also benefit from combination treatment with aminosalicylate enemas and potentially topical or even oral corticosteroids.

There are studies that have explored the benefit of higher-dose mesalazine for maintenance, and we believe that these studies add information regarding potential treatment considerations for patients with a more complex disease course, who have not achieved complete remission within 8 weeks or who relapse after dose reduction. The continued use of high-dose mesalazine is supported by a recent retrospective study that examined the efficacy of 4.0g/day maintenance therapy with mesalazine [PENTASA^®^] in Japanese UC patients who had achieved clinical improvement or remission^16^; in this study, fewer patients who received long-term [ > 105 days] vs short-term [≤ 105 days] maintenance treatment relapsed [29.8% vs 48.3%; *p* < 0.05], and the median time to relapse was also longer in the long-term treatment group. Another retrospective study comparing low- [2.4–2.8g/day] vs high-dose [4.4–4.8g/day] mesalazine maintenance treatment among UC patients found no significant differences in flare risk between groups when adherence to medication was high or moderate,^17^ suggesting that adherence may be more important for reducing flare risk than the dose used for maintenance therapy. The importance of achieving mucosal healing for long-term outcomes is supported by the results of a study by Meucci and colleagues, who demonstrated that patients achieving clinical but not endoscopic remission following 6 weeks of oral [4g/day] and topical [2g/day] mesalazine treatment experienced significantly higher cumulative relapse rates after 1 year compared with those who achieved both clinical and endoscopic remission [80% vs 23%; *p* < 0.0001].^6^

Another implication of our study findings for clinical practice is that endoscopic assessment appears to play a pivotal part in patient management, since it is an essential tool to evaluate the mucosa and guide the clinician to reach the treatment target of mucosal healing. Future mesalazine studies should explore dynamic and repeated interval dosing to allow for treatment adjustments based on long-term clinical and endoscopic stability. For example, it is unclear whether those in partial remission could benefit from a longer cycle of high-dose induction, or whether those on 2.4g/day maintenance treatment who flare could benefit from a short interval of increased mesalazine dosing post-relapse.

The safety results from both the induction and the maintenance phases indicated that multimatrix mesalazine was well tolerated. Types and frequencies of TEAEs were similar between complete and partial remission groups. The safety profile was consistent with previous multimatrix mesalazine clinical studies, and no new safety signals were identified. In previous phase 3 induction studies,^10,11^ as well as the associated maintenance study,^12^ the safety profile of multimatrix mesalazine was similar to that of the placebo arms in the induction studies.^10,11^ In the previous induction studies [combined analysis], the proportion of patients experiencing TEAEs was 32.4% for those on multimatrix mesalazine 4.8g/day, with headache being the most commonly reported [3.4%].^18^ By comparison, in the induction phase of the current study, 28.2% of patients on multimatrix mesalazine reported TEAEs, with headache again being the most commonly reported [2.1%]. In the earlier phase 3 maintenance study, 37.9% of patients experienced TEAEs, mostly of mild or moderate intensity. The most commonly reported TEAEs were worsening UC [10.7% and 7.7% in the 2.4g/day single- and divided-dose cohorts, respectively] and gastrointestinal disorders.^12^ In the maintenance phase of the current study, 37.2% of those in complete remission at Month 0 reported TEAEs, with UC [7.7%] being the most commonly reported.

Some limitations of the study include that this was an open-label study without a placebo control, which may have introduced sampling bias into the results. Also, the subjective component of the UC-DAI score may have introduced variability between investigator assessments, and subsequent classification of patients who are in endoscopic remission. Additionally, it remains unclear how previous mesalazine dosing affected the long-term outcomes of these patients, as study patients could have received up to 3.2g/day mesalazine before study entry. Finally, we did not use independent assessment of endoscopic recordings, an approach that has recently become standard in modern UC trials but was not yet common practice at the start of this study. As the same endoscopist performed both procedures in the patient, this may have introduced unconscious bias related to improvements. However, the blinding and randomisation design may have ameliorated this limitation.

The data obtained from the current study confirm that long-term maintenance with multimatrix mesalazine is safe and efficacious in patients with mild-to-moderate UC. Those who begin maintenance treatment in complete remission have improved long-term outcomes compared with those who begin maintenance treatment in partial remission.

## Funding

This work was supported by Shire Development LLC.

## Conflict of Interest

DR has received financial support for research from AbbVie, Elan, Warner Chilcott, and Prometheus; received lecture fees from Merck; and consulted for AbbVie, Janssen, UCB, Elan, Shire, Exagen, Prometheus, and Takeda. SI is a former statistical consultant for Shire, Basingstoke, UK. EM is a former employee of Shire, Wayne, PA, USA and owns stock and/or stock options in Shire. DS is a former employee of Shire, Wayne, PA, USA. GD’H has received financial support for research from AbbVie, Janssen Biologics, Given Imaging, MSD, Dr Falk Pharma, and PhotoPill; has received lecture fees from AbbVie, Tillotts, Tramedico, Ferring, MSD, UCB, Norgine, and Shire; and consulted for AbbVie, Actogenix, Centocor, Cosmo, enGene, Ferring Pharmaceuticals, GlaxoSmithKline, Janssen Biologics, Millenium Pharmaceuticals, MSD, Novo Nordisk, PDL BioPharma, Pfizer, SetPoint, Shire, Takeda, Teva, and UCB.

## Author Contributions

All authors participated in drafting of the manuscript or critical revision of the manuscript for important intellectual content, and provided approval of the final submitted version. Individual contributions are as follows. DR: study design; analysis and interpretation of data; principal investigator; guarantor. MB: patient recruitment; study site supervision. LG: patient recruitment; study site supervision; interpretation of data. DD: study site supervision. JM: patient recruitment; study site supervision; interpretation of data. SI: study design; data collection; statistical analysis and interpretation. EM: study concept and design; data analysis and interpretation. DS: study concept and design; study supervision; data analysis and interpretation. GD’H: study design; analysis and interpretation of data; principal investigator; guarantor.

All members of the Ulcerative Colitis Remission Study Group, listed below by country, assisted in patient recruitment, data collection, and study site supervision: Belgium [Filip Baert, Geert D’Haens, Francois D’Heygere]; Canada [Marc Bradette, John Marshall, Michael Ostro, Pierre Pare]; Colombia [Jacobo Feris, Fabian Juliao, Alejandro Orozco, Juan Márquez]; Czech Republic [Marek Benes, Vladimir Compel, Ladislav Douda, Libor Gabalec, Jan Hejcman, Jana Kozeluhova, Milan Lukas, Vladimir Nosek, Michal Tichy, Tomas Vanasek]; France [Arnaud Bourreille, Frank Zerbib]; Germany [Torsten Kucharzik]; Hungary [Andor Grenda, Zoltan Gurzo, Gyula Pecsi, Tibor Szaloki]; India [Rupa Banerjee, Prashant Bhandarkar, Abhijit Chandra, Bhabadev Goswami, Mukesh Kalla, Sanjay Kolte, Rupesh Bhaidas Mehta, Palakurthi Murali Krishna, Sandeep Nijhawan, K.T. Shenoy, Ajit Sood, B. Vishwanath Tantry, Vinay Thorat]; Ireland [Hugh Mulcahy, Colm O’Morain, Stephen Patchett]; Poland [Marcin Hanczewski, Marek Horynski, Robert Petryka, Wojciech Piotrowski, Jerzy Rozciecha, Marek Skoczylas]; Romania [Dan Andronescu, Christian Banciu, Daniela Dobru, Liliana Gheorghe, Adrian Goldis, Victor Stoica]; South Africa [Nazimbuddin Aboo, Frederick Bester, Suleman Moola, Maarten Prins, Christo van Rensburg, John Wright]; Spain [Javier P. Gisbert]; UK [Simon Travis]; USA [Humberto Aguilar, Richard Altman, Raj Bhandari, George Catinis, Sreenivas Chintalapani, Steven Desautels, M. Emin Donat, Michael Epstein, Syam Gaddam, Daniel Geenen, Lev Ginzburg, Lawrence Goldberg, Douglas Homoky, M. Mazen Jamal, Michael Kreines, Michael LeVine, Edward Loftus, Stephen Minton, Mark Nagrani, Vijay Narayen, Vijayalakshmi Pratha, Charles Randall, David Rubin, Peter Winkle, Douglas Wolf, Salam Zakko].

## References

[1] Cosnes J Gower-Rousseau C Seksik P Cortot A Epidemiology and natural history of inflammatory bowel diseases. Gastroenterology 2011;140:1785–94. 21530745 10.1053/j.gastro.2011.01.055

[2] Irvine EJ Quality of life of patients with ulcerative colitis: past, present, and future. Inflamm Bowel Dis 2008;14:554–65. 17973299 10.1002/ibd.20301

[3] Kornbluth A Sachar DB Ulcerative colitis practice guidelines in adults: American College of Gastroenterology, Practice Parameters Committee. Am J Gastroenterol 2010;105:501–23. 20068560 10.1038/ajg.2009.727

[4] Naganuma M Sakuraba A Hibi T Ulcerative colitis: prevention of relapse. Expert Rev Gastroenterol Hepatol 2013;7:341–51. 23639092 10.1586/egh.13.18

[5] Kane SV Systematic review: adherence issues in the treatment of ulcerative colitis. Aliment Pharmacol Ther 2006;23:577–85. 16480396 10.1111/j.1365-2036.2006.02809.x

[6] Meucci G Fasoli R Saibeni S Prognostic significance of endoscopic remission in patients with active ulcerative colitis treated with oral and topical mesalazine: a prospective, multicenter study. Inflamm Bowel Dis 2012;18:1006–10. 21830282 10.1002/ibd.21838

[7] Colombel JF Rutgeerts P Reinisch W Early mucosal healing with infliximab is associated with improved long-term clinical outcomes in ulcerative colitis. Gastroenterology 2011;141:1194–201. 21723220 10.1053/j.gastro.2011.06.054

[8] Travis SP Stange EF Lemann M European evidence-based consensus on the management of ulcerative colitis: current management. J Crohns Colitis 2008;2:24–62. 21172195 10.1016/j.crohns.2007.11.002

[9] LIALDA^®^ [mesalamine] delayed-release tablets, for oral use [package insert]. Wayne, PA: Shire US Inc; 2014.

[10] Kamm MA Sandborn WJ Gassull M Once-daily, high-concentration MMX mesalamine in active ulcerative colitis. Gastroenterology 2007;132:66–75. 17241860 10.1053/j.gastro.2006.10.011

[11] Lichtenstein GR Kamm MA Boddu P Effect of once- or twice-daily MMX mesalamine [SPD476] for the induction of remission of mild to moderately active ulcerative colitis. Clin Gastroenterol Hepatol 2007;5:95–102. 17234558 10.1016/j.cgh.2006.10.025

[12] Kamm MA Lichtenstein GR Sandborn WJ Randomised trial of once- or twice-daily MMX mesalazine for maintenance of remission in ulcerative colitis. Gut 2008;57:893–902. 18272546 10.1136/gut.2007.138248 PMC2564831

[13] Kane S Katz S Jamal MM Strategies in maintenance for patients receiving long-term therapy [SIMPLE]: a study of MMX mesalamine for the long-term maintenance of quiescent ulcerative colitis. Inflamm Bowel Dis 2012;18:1026–33. 21837775 10.1002/ibd.21841

[14] D’Haens G Sandborn WJ Barrett K Hodgson I Streck P Once-daily MMX^®^ mesalamine for endoscopic maintenance of remission of ulcerative colitis. Am J Gastroenterol 2012;107:1064–77. 22565161 10.1038/ajg.2012.103

[15] Kamm MA Lichtenstein GR Sandborn WJ Effect of extended MMX mesalamine therapy for acute, mild-to-moderate ulcerative colitis. Inflamm Bowel Dis 2009;15:1–8. 18671232 10.1002/ibd.20580

[16] Takeshima F Matsumura M Makiyama K Efficacy of long-term 4.0g/day mesalazine [Pentasa] for maintenance therapy in ulcerative colitis. Med Sci Monit 2014;20:1314–8. 25064629 10.12659/MSM.890567 PMC4136941

[17] Khan N Abbas AM Koleva YN Bazzano LA Long-term mesalamine maintenance in ulcerative colitis: which is more important? Adherence or daily dose. Inflamm Bowel Dis 2013;19:1123–9. 23514878 10.1097/MIB.0b013e318280b1b8

[18] Sandborn WJ Kamm MA Lichtenstein GR Lyne A Butler T Joseph RE MMX Multi Matrix System mesalazine for the induction of remission in patients with mild-to-moderate ulcerative colitis: a combined analysis of two randomized, double-blind, placebo-controlled trials. Aliment Pharmacol Ther 2007;26:205–15. 17593066 10.1111/j.1365-2036.2007.03361.x

